# Striving for moments of easier breathing despite being trapped in breathlessness: meanings of feeling well for women with chronic obstructive pulmonary disease stage III or IV

**DOI:** 10.1080/17482631.2023.2225937

**Published:** 2023-06-18

**Authors:** Ann Ekdahl, Siv Söderberg, Malin Holmström Rising

**Affiliations:** Department of Health Sciences Mid Sweden University, Sundsvall, Sweden

**Keywords:** Chronic obstructive pulmonary disease, feeling well, health, lived experience, individual interviews, phenomenological hermeneutic method, qualitative method, women’s health

## Abstract

**Background:**

Living with chronic obstructive pulmonary disease stage III or IV means living an everyday life, severely restricted by breathlessness.

**Aim:**

The aim of this study was to elucidate meanings of feeling well for women with chronic obstructive pulmonary disease stage III or IV.

**Method:**

The study has used a phenomenological hermeneutical design. Individual narrative interviews were conducted with 14 women with chronic obstructive pulmonary disease at stages III or IV.

**Results:**

The results revealed one theme: striving for moments of easier breathing despite being trapped in breathlessness with four subthemes: acting in rhythm with breathing, taking care of oneself, taking advantage of better moments, and being in togetherness in everyday life.

**Conclusion:**

This study shows that women with chronic obstructive pulmonary disease at stages III or IV strived for moments of feeling well despite living with a severe illness. Feeling well meant that when connected to nature, they felt alive, free, and less trapped in breathlessness, which provided a sense of being unconscious of their breathing rhythm. They could do what healthy people tend to take for granted during everyday life. To feel well, the women found it important to receive tailored support from their close relatives.

## Introduction

Living with a long-term illness such as chronic obstructive pulmonary disease (COPD) at stages III or IV means living an everyday life that is severely restricted by fluctuating breathlessness (Ek et al., 2011; Global Initiative for Obstructive Pulmonary Lung Disease; (GOLD), 2023). Previous research has shown that people with COPD experience breathlessness as the most common symptom permeating their lives. Respiratory difficulties such as COPD are complex and have multifactorial influences on everyday life as well as on the patients’ well-being (Ferreira et al., 2020; Miravitlles et al., 2007; Pesola & Ahsan, 2016; Sandberg et al., 2019). Globally, COPD is forecast to become the third-leading cause of death by 2030; during recent years, its prevalence among women has equalled that of men in many high-income countries (WHO, 2022); GOLD (2023).

Everyday life with limitations caused by COPD can lead to constant worry regarding being able to function normally, move, walk, or sometimes even speak. It affects both the sufferer and their close relatives (Ali et al., 2017; Ferreira et al., 2020). Depression and anxiety are common, affecting 55% of people who have COPD worldwide (Willgoss & Yohannes, 2012). Further, increased risk of exacerbation and subsequent hospitalization seems to further increase the risk of mortality, while health-related quality of life is reduced due to depression and anxiety (Kong & Wilkinson, 2020). Prior studies report impaired quality of life and reduced health-related quality of life for both men and women with COPD (Jerpseth et al., 2021; Miravitlles et al., 2009; Szentes et al., 2020). For people with COPD, good days alternate with bad days (Giacomini et al., 2012; van der Meide et al., 2020). In addition, women are more likely to bear a heavier share of the burden of symptoms of COPD than men (Aryal et al., 2014; Buttery et al., 2021; GOLD, 2023). Research on the perspectives of women’s experiences with COPD indicates that they face multiple prejudices and stigmas, and a frequent lack of knowledge (GOLD, 2023; O’Neill, 2002; Rose et al., 2017; Steindal et al., 2017). Women with other chronic illnesses have described how they can feel well even though they live with a long-term illness such as fibromyalgia or multiple sclerosis (Juuso et al., 2013; Olsson et al., 2008). Stridsman et al. (2015) noted that people with COPD at stages II—IV experience good days and increased well-being at times when they can breathe and when they experience a balance between breathing and coping. Altered well-being and women’s sexual dissatisfaction are often underestimated in people with COPD (Zysman et al., 2020).

Some prior research indicates that women with COPD at stages III—IV were stabilizing an ever-present breathlessness by restoring strength to manage everyday life and adapt to their limited abilities and energy (Ekdahl et al., 2021). Breathing is natural for healthy people; when breathing becomes a hindrance, however, nothing else matters (Similowski, 2018). People with healthy bodies experience their bodies as absent, meaning that healthy people tend not to be aware of bodily functions, such as the processes of our inner organs or the anonymous breathing that keeps us alive (Leder, 1990; S. K. Toombs, 1995). Breathlessness is a subjective experience and a lived symptom; the sensation is affected by oxygen saturation level, respiratory effort, chest muscle work, and blood pH level. When oxygen desaturation is severe, feelings akin to suffocation, dizziness, loss of control, and incontinence are often present. Healthcare professionals show a tendency to stick to objective physiological measurements, mainly due to the limited understanding in the field of the subjective nature of complex breathlessness (Carel, 2016; Carel et al., 2015).

To the best of our knowledge, there is no previous research about meanings of feeling well for women who have COPD at stages III or IV. Knowledge regarding what it means to feeling well for women with COPD is a prerequisite for providing support that aligns with their needs and resources, and can form a foundation to promote their health and well-being. Therefore, the aim of this study is to elucidate meanings of feeling well for women with COPD at stages III or IV.

## Materials and methods

This study utilizes a phenomenological hermeneutic method inspired by the French philosopher Ricoeur’s (1976) interpretive theory, which relates the life-world theory of phenomenology to a hermeneutical focus on interpretation and understanding (Lindseth & Norberg, 2004). Lindseth and Norberg developed this method for nursing research, striving to gain a deeper understanding of the phenomena under study. The choice of this method was motivated by the aim to elucidate meanings of the phenomenon of feeling well for women with COPD at stage III or IV.

## Participants and procedure

A purposive sample of of 14 women with COPD stage III or IV participated in this study. The participants were recruited with assistance from a coordinator at a hospital in central Sweden; the coordinator distributed 50 invitation letters in line with the inclusion criteria described below. Fourteen women agreed to participate; each received information about the study, an informed consent form, and a reply form. They sent their informed consent to the first author. The first author then contacted the 14 women by telephone, to schedule individual interview appointments. Before starting the interviews, each participant was given oral information, highlighting the voluntary nature of their participation. Each participant was provided time to ask questions and information to contact the first author later, if needed.

The inclusion criteria were as follows: female participants aged over 18, with a diagnosis of COPD at stage III or IV, who had the ability to speak and understand Swedish. The 14 participants were aged between 52 and 86 years (md = 69 years) and had been diagnosed with COPD between 2 to 25 years previously (md = 16 years). Seven women were receiving oxygen treatment at home. Two had alpha−1 antitrypsin deficiency. Three had a secondary school education, seven had completed high school., and two had some higher education. Twelve of the women were retired or had taken early retirement; one had a part-time job, and one was unemployed. Five lived alone, while nine were married or lived with a partner. Most of the women lived at home in a flat, or apartment, and a few lived in a house. Four women had home-care services. The participants lived in rural and urban areas of the region.

## Interviews

Individual narrative interviews were conducted with the participants (cf. Mishler, 1986). Narratives are stories persons tell about their lived experiences specific to the aim. The interviews elucidated various meanings of feeling well for women with COPD at stage III or IV. The stories described the women’s past, present, and future (Mishler, 1986). The interviews began with an opening question, with the women asked to talk about their experiences of feeling well and your experiences when you are feeling less well. Follow-up and encouraging questions were also asked, such as “What do you mean?”; “Who said or did what?”; “What happened next?”; and “Can you tell me more about that?” The interviews lasted between 30 and 70 minutes (md 57), were digitally recorded, and were transcribed verbatim. The interview texts were rich and representative, which enhanced information power (Malterud et al., 2016). The interviews were conducted by the first author from March to October 2022 by telephone, as the choice of all participants. The first author is a registered nurse with previous clinical experience in meeting people with severe long-term illnesses.

Follow-up interviews were conducted with 11 women one to two weeks after the first interview. The aim of these follow-up interviews was to give the women an opportunity to add information, make corrections, and ask clarifying questions if needed.

## Context

The study was performed in the central region of Sweden, which has a population of approximately 245,000 inhabitants in an area of 21,548 km^2^, including both rural and urban areas (Statistics Sweden, 2023; Swedish Agency for Economic and Regional Growth, 2022). In Sweden, the population who have any stage of COPD is between 500,000 and 700,000 people; for stages III and IV, the prevalence is approximately 7% (Backman et al., 2016; Lindberg et al., 2006).

## Data analysis: the phenomenological hermeneutic interpretation

The interview texts were analysed with a phenomenological hermeneutic interpretation developed by Lindseth and Norberg (2004). This method implies a process of interpretation containing three phases, characterized by movement between the whole and the parts of the interview texts, between understanding and explanation, and ending with new comprehension (Lindseth & Norberg, 2004). All authors collaborated on the analysis, having illuminative discussions until a mutual understanding was reached. The process started with a naive reading, which describes a first understanding of the whole text after several readings of the interview text. This was followed by the structural analysis, in which the texts were divided into units of meaning. The meaning units were then condensed and sorted into subthemes and themes. The interpretation of the naive reading needed to be either validated or invalidated by following the structural analysis (Lindseth & Norberg, 2021). Table 1 provides an example of the structural analysis. The last phase of analysis was the comprehensive understanding, in which the text was again interpreted as a whole, based on the authors’ initial understanding, the naïve understanding, the structural analysis, and literature (cf. Lindseth & Norberg, 2004).

**Table 1.** Example of structural analysis.

Meaning units	Condensed meaning units	Subthemes	Theme
*I take it easy and think about how I breathe and act. That’s my life nowadays … I have the whole day. That’s how I endure … I feel well if it works. (P9)*	Take it easy and think about how to breathe and act to feel well	Acting in rhythm with breathing,	Striving for moments of feeling well despite being trapped in breathlessness
*I got a walker to avoid carrying the oxygen device going outdoors… I can sit down and rest from time to time if I have a walker. I go to the store and run errands and things like that independently, but I do not go long distances. (P1)*	Got a walker to carry the oxygen and to rest to go to the store and run errands independently	Taking care of oneself	
*It’s just a matter of taking the opportunity of those better moments … do these things that everyone takes for granted like having a cup of friendly coffee… I try to reflect, and I just roll on and hang around … When you live a life like me, you should have a little fun instead [laughs]. (P5)*	Taking opportunities of better moments when you live a life like me	Taking advantage of better moments	
*I have my great-grandchildren I’m knitting for … and I talk to my children almost every day and… I have a rich life. (P10)*	Have my children and grandchildren and talk to them and have a rich life	Being in togetherness in everyday life	

## Ethical considerations

This study followed and was conducted in accordance with the Declaration of Helsinki, which supports respect for all human beings and protection of their health and rights when participating in research (World Medical Association, 2018). The Ethical Review Agency in Sweden (Dnr: 2022–00369–02) has approved this study. All participants received oral and written information about the study before the interviews, and all signed a consent form, which is securely stored by the first author. The participants were informed that their participation was voluntary, and that they could withdraw at any time without any explanation. They were guaranteed confidentiality and assured that their anonymity would be preserved in the presentation of the findings.

## Results

### Naïve understanding

Feeling well for women with COPD at stage III or IV seemed to be embodied in moments of less breathlessness and fewer physical limitations. Feeling independent and managing everyday life seemed important to moments of feeling well, but demanded planning and was orchestrated around the women’s breathing rhythm. Moments of feeling well seemed to be dependent on the women’s ability and knowledge of breathing and body limitations. A woman’s use of her energy enabled endurance and seemed to create moments of feeling well. Close relatives or friends appeared to be fundamental to supporting such times of feeling well for women with COPD at stage III or IV. Digital solutions seemed to create moments of feeling well, by enabling communication and presence across distances. Relationships to others appeared to create feelings of togetherness and worthiness, and senses of contributing to society or to family; these relationships appeared to be important contributors to moments of feeling well for women with COPD stage III or IV.

### Structural analysis

The structural analysis revealed one theme with four subthemes. The theme was *striving for moments of easier breathing despite being trapped in breathlessness*, with four subthemes: *acting in rhythm with breathing*, *taking care of oneself*, *taking advantage of better moments*, and *being in togetherness in everyday life*. The following text presents the results with referenced quotes from the anonymized participants (P1, P2 …).

#### Striving for moments of easier breathing despite being trapped in breathlessness

##### Acting in rhythm with breathing

Based on their experience and knowledge of breathing and not being afraid of breathlessness, women with COPD at stage III or IV planned for moments of feeling well by acting in rhythm with their breathing. Being forced to act in rhythm with their breathing meant that sometimes they could manage breathlessness and find sufficient energy to accomplish what they wanted; thereby, they arrived at feeling well. Women were acting in rhythm with breathing to endure being trapped in breathlessness. Persevering with a positive attitude kept negative feelings and thoughts at bay; the women expressed that this was a way to enhance their motivation to feel well and recognize their feelings, just as when they had been healthy. When participants were able to follow their planned structure during everyday life, for example planning a meal, reading, walking the dog, cuddling the cat, knitting, or watching TV, this meant feeling well.

*I take it easy and think about how I breathe and act. That’s my life nowadays … I have the whole day. That’s how I endure … I feel well if it works.* (P9)

*I know what to eat, how to do energy-saving measures and how to breathe to feel as well as I can … if I get heavy breathlessness.* (P8)

They admitted that being able to do what they wanted was difficult, considering they often lacked the energy to do so; times when this was the case were followed by feeling less well.

Acting in rhythm with breathing and being able to go outdoors were desirable for women with COPD at stage III or IV. Spending time in nature meant feeling well as a positive influence. Going out to the pavement or sitting by their doorstep breathing in the fresh air mediated the reconnection of their bodies, meant feeling well. These moments in nature meant experiences of feeling joy, peace, freedom, relaxed, and longing for the experience of nature and being alive. In this context, memories of being able and connected to nature seemed to be an important drive awakening the senses and the desire to experience nature; these in turn seemed connected to self-confidence and feeling well.

*I’m very much in favour of being out in nature and in the woods, but now I can’t do it much anymore … It means a lot … just to sit down on a stump and just suck in air and everything that’s around … I really feel that I’m alive … Out in nature, I feel free from all the misery*. (P3)

When participants felt themselves trapped in breathlessness, this meant counteracted or undermined the times when they were feeling well. Times when they were unable to do what they wanted to—such as socializing with friends, dancing, or baking—due to their inability to acting in rhythm with breathing was frustrating.

#### Taking care of oneself

Feeling well for women with COPD at stage III or IV meant managing and taking care of themselves in everyday life by relying on various types of support. Taking care of oneself meant that physical aids were important and enabled them to feel independent. The physical aids they described meant that using a walker to go out for a stroll, using a shower chair to manage hygiene, or using an adjustable bed to sleep more comfortably. Women in need of oxygen treatment described being able taking care of themselves to a greater extent by using portable oxygen.

*I got a walker to avoid carrying the oxygen device going outdoors … I can sit down and rest from time to time if I have a walker. I go to the store and do errands and things like that, but I do not go long distances.* (P1)

The support of close relatives meant feeling well when it resulted in that women with COPD at sage III or IV were taking care of themselves, such as helping to reach things they needed that were high up or too far away. Close relatives acted as an extension of the women themselves in everyday life, thereby supporting participants’ senses of independence and feeling well. For the women, taking care of themselves by being physically mobile meant feeling well and slowing down the progression of the illness. The support obtained via suitable tools and alternative approaches that enabled them to be physically mobile meant that knowledge of how to perform activities was important. Alternatives that contributed to feeling well might include walking indoors, utilizing simple movements in the kitchen, and participating in a TV-led workout which also meant feeling well.

*I’ve a tread mill in house that I use every single day. I can use oxygen in a little bag when I get on the walking band … I do it to stay feeling well.* (P6)

*I would like to have a parking permit because then I would be able to park my car and do errands myself … You become a bit dependent on other people if you say so*. (P14)

Taking care of oneselves for a lifetime also includes the end-of-life stage. To the women, this seemed to mean it was easier to prepare for the end when they could obtain further knowledge, take part in discussions with close relatives, and have the ability to express thoughts and fears about end-of-life issues to healthcare professionals.

*People can write and sign some papers before you get really, really ill, about how you would like the end to be … I want these papers prepared before I get so ill that I can’t communicate … and the kids know about it beforehand. That way I won’t have to be afraid of suffocating and I will be more content*. (P7)

#### Taking advantage of better moments

Taking advantage of better moments meant that women with COPD at stage III or IV seemed to live in the present, despite the limitations of their breathlessness. They saw these moments as highly valuable, because they felt their time was running out. Feeling well in this context included moments of reflecting, enjoying being alive, and being able to perform meaningful tasks. To take advantage of these better moments, participants wanted to perform such actions as shopping for groceries and, if they had enough strength, perhaps meet people for coffee or take care of some housekeeping. They wanted to do what healthy people tend to take for granted, and have a bit of fun in life.

*… it’s just a matter of taking the opportunity of those better moments … do these things that everyone takes for granted like having a cup of friendly coffee … I try to reflect, and I just roll on and hang around … When you live a life like me, you should have a little fun instead. [laughs]* (P5)

Taking advantage of better moments involved making use of time; this seemed to boost the confidence of women with COPD stage III or IV, through their knowledge of breathing, thereby contributing to their feeling well. They expressed a belief that bodily limitations and fluctuating breathing meant accepting that ordinary things took time while they felt trapped in breathlessness. Managing everyday life and taking slow walks without speaking could comprise an entire day’s project; the participants saw these as highly valuable. The participants cherished and took advantage of moments when they felt well; for them, a reward was sitting relaxed, feeling content, quietly eating fruit on the couch, and feeling unaware of their breathing rhythm.

*In moments when I experience feeling well is at eight thirty in the evening … I have filled my little enamel bowl with fruit sitting relaxed by the television*. (P12)

To hurry was impossible for women with COPD stage III or IV and it was important to them that close relatives share this understanding.

#### Being in togetherness in everyday life

Being in togetherness in everyday life with family and friends meant that feeling well was possible when the women with COPD at stage III or IVs breathing was easier. Being in togetherness was important, it was the meaning of life itself, and gave purpose to women’s lives. They managed to maintain contact with family and friends by using telephones, digital communication, and social media, in addition meeting face-to-face, all of which contributed to moments of feeling well. Through these means, the distant presence of close relatives became possible; the long-distance communication was crucial to participants avoiding being alone and enhancing feeling well. Being able to provide some care for their relations contributed to feelings of worthiness as equals among the women with COPD stage III or IV. They only needed to use their ability to speak in order to feel more well.

*… I talk to my son on the other side of the world … He calls me up digitally … I talk to him and then he talks to me. We can talk for about maybe half an hour a day in the morning, almost every day … We keep an eye on each other and that makes me feel well, and that’s great*. (P4)

*I have my great-grandchildren I’m knitting for … and I talk to my children almost every day and … I have a rich life.* (P10)

Meaning of feeling well for women with COPD stage III or IV involved feeling worthy through contributing to the lives of their family and friends. During moments when they had capacity, they contributed in various ways, including working in a board committee, actively engaging in patient associations and COPD groups on social media, or babysitting grandchildren. These activities generated feelings of togetherness for participants; in contrast, when they felt trapped in breathlessness, they described a lost sense of being able to care for others, or a lost sense of being able to contribute as worthy and active members of society. As women with COPD at stage III or IV, they often needed help and support, and feeling excluded generated feelings of dependence. Inability to contribute economically to their families due to their bodily limitations meant feelings of frustration and of dependence. Being in togetherness and feeling loved by family, feeling appreciated for who they were as people, and visits from family or friends all contributed to feeling well and the sense of a rich life among the participants.

*You don’t just want to be like a parasite … You want to be able to do something … to contribute. Then I feel well.* (P9)

## Comprehensive understanding and reflections

During the last phase of the interpretation process, the interview texts were viewed as a whole. The naïve understanding, the structural analysis, and the authors’ pre-understanding were brought together into a comprehensive understanding, which was then reflected on (cf. Lindseth & Norberg, 2004). The results from this study will be interpreted in line with the work about the illness experiences presented by Carel (2016, 2021), K. Toombs (1993) and prior research. The choice of Carel and Toombs is based on their research; Carel about the phenomenon of breathlessness, and Toombs the meaning of illness.

The findings show that feeling well for women with COPD stage III or IV, meant that they strived for moments of easier breathing despite being trapped in breathlessness. Breathing is what people normally take for granted; according to Carel (2021), breathlessness is an extreme experience of breathing. According to Ekdahl et al. (2021) women with COPD stage III or IV lived with an ever-present breathlessness and did not take their breathing for granted.

Women with COPD at stage III or IV planned for the moments when they would feel well. When acting in rhythm with breathing, they were able to perform tasks they wanted to complete, although these remained difficult. For participants, feeling well was enhanced by maintaining motivation, keeping a positive attitude, and recognizing when they felt well. This enabled them to independently make plans to perform tasks that were important to them. Carel (2021) has expressed that if a person’s breathing is in harmony and in rhythm with their body energy, this could be seen as an experience of feeling well despite living with a serious illness. In addition, Cooney et al. (2013) have described the creativity people with COPD stage III or IV utilize to make plans during their “battle” to continue to live life on their own terms despite their breathing limitations.

In the current study, participants saw spending time in nature as highly valuable. Connecting to nature caused them to feel alive, free, and less trapped in breathlessness. These moments meant feeling well, and thereby they could experience easier breathing. This can be seen as what K. Toombs (1993) expressed as the body’s here-ness: people cannot physically distance themselves from their bodies; thus, they may separate themselves from certain parts of their bodily reactions under the right circumstances.

In addition, feeling well for the women with COPD stage III or IV who participated in the current study meant striving to independently take care of themselves as much as possible; this appeared to be key. Physical aids were vehicles that enabled them to reach a wider world. Carel (2012) has noted that bodily limitations can be overcome by physical aids when they are integrated with part of the body. It appeared that women needing oxygen treatment could use assistive devices to take care of themselves to a greater extent; thus, this contributed to them feeling well. This is in line with Ek et al. (2011), who have argued that people utilizing oxygen treatment for severe COPD experience increased freedom in everyday life.

Having the support of close relatives was important for women with COPD stage III or IV to feel well; this type of family interaction functioned as an extension of participants, which increased their capacity to take care of themselves. Carel (2016) argues that breathlessness changes the geography of a person’s everyday life. Within this changed geography, via tailored support from close relatives, the participants in the current study were able to feel well.

The participants also were able to feel well when being physically mobile; this sometimes included stabilizing the progression of their illness. They had knowledge of how to perform activities and be creative. Carel (2007) has noted that finding a new way of performing an old task through physical activity leads to a sense of achievement. The participants knew time was running out and sought to live in the present and take advantage of better moments. According to Carel (2009), fully concentrating on the present is an important part of feeling well. For women with COPD at stage III or IV, feeling well meant doing what healthy people tend to take for granted, such as reflecting on the joy of life.

Our results indicate that in order to feel well, women with COPD stage III or IV accepted that ordinary tasks took a longer time to complete. The participants experienced feeling well when they were unconscious of their breathing rhythm; for example, when they felt relaxed, they were content. This aligns with Carel (2016), who describes a sensation of breathing unstrained and pleasant as when relaxed.

This study indicate that being in togetherness and having positive relationships meant feeling well for participating women with COPD at stage III or IV, and appeared to be the meaning of life itself. Making contributions to family, close relatives, and society in togetherness meant feeling worthy and thereby feeling well for the participants. Bolton et al. (2022) argue that if some sense of meaning and purpose remains present in the everyday life of people with severe COPD, such as still being able to identify their contributions to society, life is given a purpose.

## Methodological considerations

The aim of this study was to elucidate meanings of feeling well for women with COPD at stage III or IV. The phenomenological hermeneutical method involves elucidating meanings of life-world phenomena (Lindseth & Norberg, 2021). In this study, we have found that women with COPD stage III or IV were striving for moments of easier breathing despite being trapped in breathlessness.

The sample was purposive and heterogeneous, and consisted of 14 women with experiences of the phenomenon under study. Data were obtained through individual narrative interviews. The interviewer strove to be attentive and flexible, and the interviews were guided by an opening question that mirrored the aim of the study. The participants shared rich descriptions of their lived experience of feeling well; therefore, we found the sample size sufficient to the aim of the study. The results of this study can be transferred to similar situations if the results are recontextualized to the new contexts (cf. Lindseth & Norberg, 2004). According to Ricoeur (1976), there is more than one way to interpret a text. The interpretation not only must yield the probable explanation, but also must be more likely than any other interpretation. The presented interpretation in this study is the one we found to be most probable and likely.

## Conclusion

This study shows that women with COPD at stage III or IV strived for moments of feeling well despite living with severe illness. For these women, feeling well meant that connecting to nature made them feel alive, free, and less trapped in breathlessness. In addition, with tailored support from close relatives, women with COPD stage III or IV could feel well. For the participants, feeling well also meant being able to do what healthy people tend to take for granted. The women also experienced feeling well when unconscious of their breathing rhythm, such as when they felt relaxed. It is important to understand what contributes to feeling well, such as women’s expertise and experiences of living with COPD stage III or IV. If the women were listened to and were provided the chance to influence their own care, they could experience additional opportunities to experience moments of feeling well.
